# Natural history of Duchenne muscular dystrophy in the United Kingdom: A descriptive study using the Clinical Practice Research Datalink

**DOI:** 10.1002/brb3.3331

**Published:** 2023-11-13

**Authors:** Jonathan Broomfield, Keith Abrams, Nick Latimer, Michela Guglieri, Mark Rutherford, Michael Crowther

**Affiliations:** ^1^ Department of Population Health Sciences University of Leicester Leicester UK; ^2^ Department of Statistics University of Warwick Coventry UK; ^3^ Centre for Health Economics University of York York UK; ^4^ School of Health and Related Research (ScHARR) University of Sheffield Sheffield UK; ^5^ John Walton Muscular Dystrophy Research Centre Newcastle University and Newcastle Hospitals NHS Newcastle UK; ^6^ Red Door Analytics Stockholm Sweden

**Keywords:** Duchenne muscular dystrophy, economic models, electronic health records, statistical models

## Abstract

**Background:**

Duchenne muscular dystrophy (DMD) is a rare, muscle‐degenerative disease predominantly affecting males. Natural history models capture the full disease pathway under current care and combine with estimates of new interventions’ effects to assess cost‐effectiveness by health technology decision‐makers. These models require mortality estimates throughout a patient's lifetime, but rare disease datasets typically contain relatively few patients with short follow‐ups. Alternative (published) sources of mortality data may therefore be required.

**Methods:**

The Clinical Practice Research Datalink (CPRD) was evaluated as a source of mortality and natural history data for future economic evaluations of health technologies for DMD and rare diseases in general in the UK population. This retrospective longitudinal cohort study provides flexible parametric estimates of mortality rates and survival probabilities in the current UK DMD population through primary/secondary records in the CPRD since 1990. It also investigates clinically significant milestones such as corticosteroid use, spinal surgery, and cardiomyopathy in these patients.

**Results:**

A total of 1121 male patients were included in the study, observed from 0.7 to 48.9 years. Median life expectancy was 25.64 years (95% confidence interval 24.73, 26.47), consistent with previous global estimates. This has improved to 26.47 (25.16, 27.89) years in patients born after 1990. The median ages at corticosteroid initiation, spinal surgery, ventilation, and cardiomyopathy diagnosis were 6.06 years (5.77, 6.29), 14.79 years (14.29, 15.09), 16.97 years (16.50, 18.31), and 15.26 years (14.22, 16.70), respectively.

**Conclusions:**

Estimates of mortality in UK‐based DMD patients are age‐specific in a uniquely large and nationally representative sample from the CPRD.

## Graphical abstract

This study evaluated the Clinical Practice Research Datalink (CPRD) as a source of mortality and natural history data for Duchenne Muscular Dystrophy (DMD) in the UK population. This can help facilitate future economic evaluations of health technologies for DMD and rare diseases in general. Median life expectancy was consistent with previous global estimates, while secondary clinical outcomes were underreported.

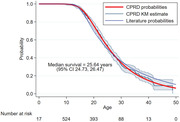

## 1 INTRODUCTION

Duchenne muscular dystrophy (DMD) is a rare, degenerative disease with a global prevalence of 1 in 3500−5000 live male births (San Martin & Solis, 2018) and 1 in 50 million live female births (Nozoe et al., 2016). DMD is a multi‐faceted disease with key milestones relating to loss of ambulation, loss of the ability to transfer, loss of upper body functions, spinal surgery for scoliosis, ventilator use, cardiomyopathy, and mortality. No cure exists for DMD, and many therapeutics are being explored in clinical trials, predominantly focusing on prolonging ambulation, but also helping to reduce the impact of other aspects of DMD. The current standard of care and a proactive, multi‐disciplinary approach including oral corticosteroids, assisted mechanical ventilation, and early initiation of cardiac medication at first signs of cardiomyopathy (Birnkrant et al., 2018) delays the loss of ambulation and prolongs life expectancy.

Once the safety and efficacy of a new treatment have been demonstrated, companies then need to prove to funding organizations such as the National Institute for Health and Care Excellence (NICE) that it is cost‐effective. This requires accurate modeling of the disease natural history. The model must demonstrate that the incremental costs and effects of a newly developed intervention in the treatment pathway offer better value than approved, standard care. In particular, this involves incorporating mortality in order for quantities of interest (such as quality‐adjusted life years) to be derived (NICE, 2016). This can prove challenging as clinical trials investigating treatments for DMD typically last/have primary endpoints measured at a relatively short period of time compared to the natural history of the disease and have predominantly been in younger patient cohorts where mortality rates are lower (Broomfield et al., 2021).

Mortality in patients with DMD has generally been published in isolation, representing a single practice or geographical area (Eagle et al., 2002; van den Bergen et al., 2014). The Cooperative International Neuromuscular Research Group has conducted an international natural history study on 440 patients with DMD (ClinicalTrials.gov, 2005; McDonald et al., 2018), but to date, no mortality results have been published. Though some long‐term studies are ongoing, prospective natural history modeling of DMD for future economic models is complicated by the rarity of and sparseness of long‐term follow‐up in the disease area. Landfeldt et al. (2014) instead estimated the economic burden of DMD via a cross‐sectional study, but this is susceptible to recall bias and may not be generalizable. Recent work by Project HERCULES with the Critical Path Institute (Critical Path Institute, 2016) has estimated a full natural history model through registry data, clinical trial control arms, elicitation data, and published mortality data (Broomfield et al., 2023).

Life expectancy for patients with DMD has been estimated at 25.3 years (95% confidence interval 23.1, 26.6) (Eagle et al., 2002), while in more recent years, this has increased to around 31.7 (27.4, 36.0) in some populations (Wang et al., 2018), and many studies note trends of increasing life expectancy with time (Ishikawa et al., 2010; Kieny et al., 2012; San Martin & Solis, 2018; van den Bergen et al., 2014). Systematic reviews by Broomfield et al. (2021) and Landfeldt et al. (2020) have estimated median life expectancy to be between 28.1 and 29.9 years in current DMD populations.

This study is conducted to estimate age‐specific mortality rates and provides a descriptive overview of the UK male population of patients with DMD from population linked electronic health records. Data were extracted from the Clinical Practice Research Datalink (CPRD), representing a uniquely large cohort of UK‐based patients with DMD. The CPRD contains longitudinal primary and secondary care records of patients attending a sample of UK‐based general practices, which patients with DMD attend alongside specialist center visits, and is split into GOLD and Aurum. The only difference between the two databases is in the software used to store anonymized patient records (Wolf et al., 2019). Crucially, the CPRD provides a sample of patients based solely pon the fact that they are registered with a CPRD practice, not selected conditional on attending a specialist center. Therefore, these rates can be used in future economic modeling and health technology assessments of new treatments for DMD in the United Kingdom as they are representative of the current UK population. The disease progression of this patient population in relation to corticosteroid use, spinal surgery, ventilation, and cardiomyopathy was also investigated. Finally, the study evaluates the use of registry data as an alternative, less costly source of mortality data for rare diseases.

## 2 METHODS

Male patients from both GOLD and Aurum were extracted to maximize numbers. Details on the extraction process are available in the Appendix. A feasibility count in CPRD GOLD identified 458 male patients, and a count in CPRD Aurum identified 985 male patients. Since there are typically around 2500 males with DMD living in the United Kingdom at any one time (National Health Service, 2022), this study represents a significant portion of patients with DMD.

The recruitment period was the beginning of CPRD collection to the date of extraction (summer 2021). Patient follow‐up began at their date of birth. Exit time was first defined as the date of death as recorded by the Office for National Statistics (ONS), or if this was missing CPRD death date. For patients who had no recorded date of death, exit time from the study was defined as the latest follow‐up date from their CPRD records and Hospital Episode Statistics (HES) records before the extraction date.

To negate the possibility of misdiagnosed patients (e.g., patients with a DMD medcode that had a milder dystrophy, such as Becker Muscular Dystrophy), any patients with a record observed at an age greater than 50 were removed from the final analysis. This assumption was investigated in a sensitivity analysis and is consistent with previous studies of mortality in patients with genetically confirmed DMD diagnoses (Broomfield et al., 2021). The analysis was also rerun on patients with at least two DMD medcodes, and on patients with an ICD‐10 code of G71.0 (Muscular Dystrophy) appearing in their HES diagnosis records, to represent populations with a more concrete DMD diagnosis.

Survival regression was used to estimate age‐specific mortality rates, with age as the timescale and modeled using splines. This approach provided smooth estimates of survival probabilities and mortality rates across the disease pathway. Analysis follow‐up time was restricted to post‐1990 so that only follow‐up after this date contributed to the analysis, increasing data quality and allowing more up‐to‐date estimates of survival to be obtained (Keogh et al., 2018). Further restrictions of follow‐up time to post‐2000 and then post‐2010 were investigated. Patients were also left‐truncated at the date of registration at a patient's practice to avoid immortal‐time bias (Tyrer et al., 2022). This also negates the possibility of patient duplication across GOLD/Aurum, since age‐specific rates are conditional only on patients currently contributing to follow‐up at each time point.

Secondary outcomes of first corticosteroid use (Bushby et al., 2010a, 2010b), spinal surgery (Eagle et al., 2007), ventilation, and cardiomyopathy (Spurney, 2011) were also extracted from CPRD/HES records as these are significant clinical milestones in DMD. First corticosteroid use, rather than temporal use, was investigated as the consistency of steroid records was unclear. Some patients had large gaps of up to 5 years between steroid records, which is unlikely to reflect their actual steroid profile over time. Steroid users in this study are therefore defined as patients with at least one prescription of corticosteroids in their records. The full lists of corticosteroid codes, with details on doses and molecules, from CPRD GOLD and Aurum are available in the Appendix, alongside code lists for spinal surgery, ventilation, and cardiomyopathy. Patients are only under observation for the proportion of their lives that they are visiting a CPRD practice, and if they experience events outside of this window, this will not be recorded. A sub‐analysis compared the breakdown of secondary events by pediatric and adult populations (over/under 18 years).

To investigate temporal changes in survival and secondary events (corresponding to improved care and disease management through steroid use, mechanical ventilation, and early cardiac interventions), calendar year of birth was included as a covariate, categorized into patients born before and after 1990.

Analyses were restricted to patients at practices eligible for linkage to ONS mortality and HES data. A sensitivity analysis relaxed this to include all patients identified by the DMD medcodes. Full sensitivity analysis results are given in the Appendix.

## 3 RESULTS

### 3.1 CPRD overview

**FIGURE 1:**
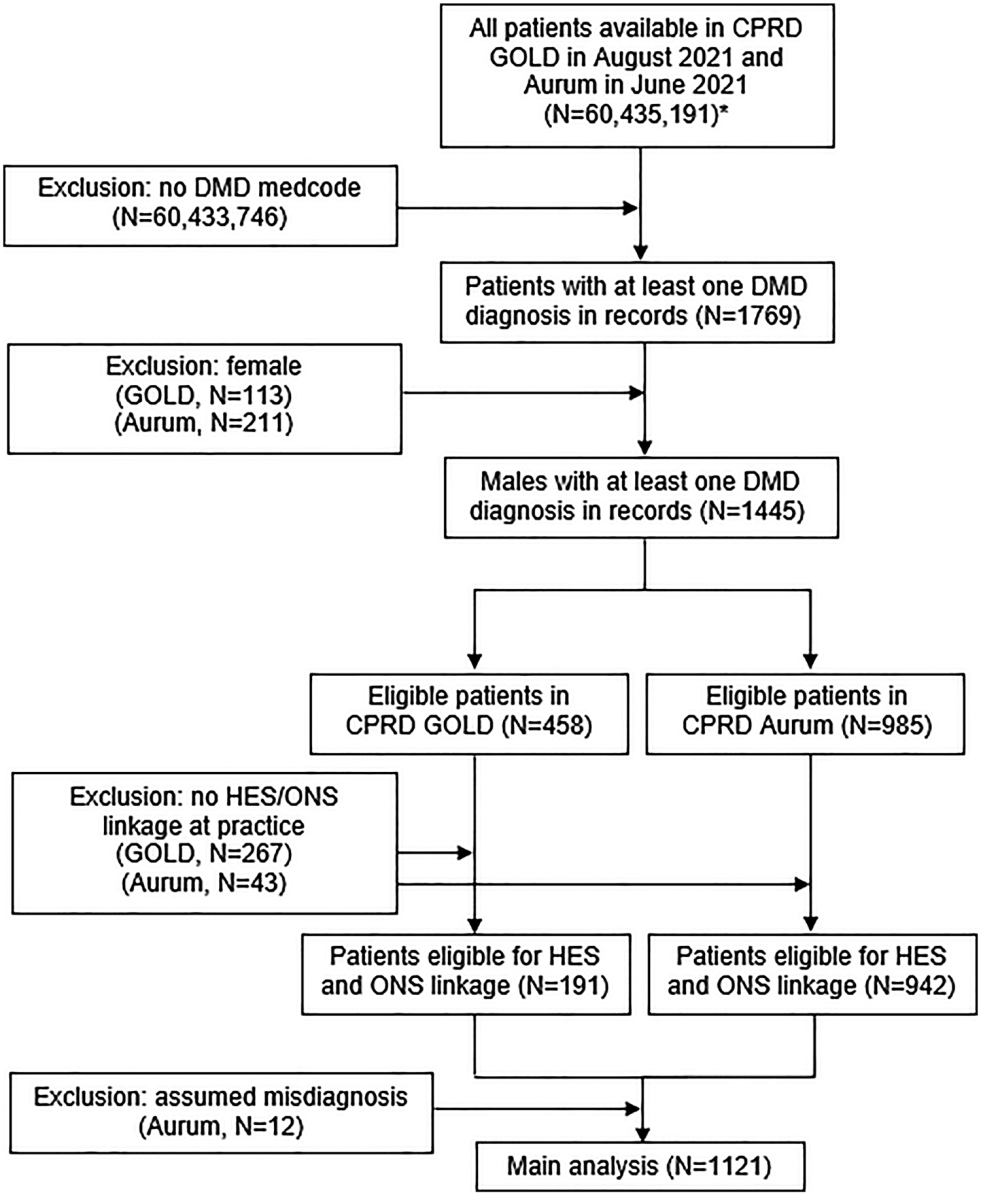
Flow of patients in the study. ^*^Numbers taken from Clinical Practice Research Datalink (CPRD) website (CPRD GOLD, 2021; CPRD Aurum, 2021). HES, Hospital Episode Statistics; ONS, Office for National Statistics.

The flow of DMD patients in this study is shown in Figure 1.

While 324 female patients were identified by the initial DMD diagnosis, it is likely that these patients are merely carriers of DMD since the disease is X‐linked. Approximately 96% of the 985 patients in CPRD Aurum with a DMD medcode were eligible for HES linkage, compared to just 42% of the 458 patients in CPRD GOLD (discussed further below). In total, N = 1121 patients were included in the final analysis (337 of whom had died). The average follow‐up per patient was 10.6 years, with an average of 8.9 years of follow‐up in patients before the age of 18 and 6.0 years after.

The breakdown of patients and practices by GOLD and Aurum is presented in Table 1.

**TABLE 1:** Number of practices and patients diagnosed with Duchenne muscular dystrophy (DMD) registered in Clinical Practice Research Datalink (CPRD) GOLD/Aurum.

	CPRD GOLD		CPRD Aurum		Total	
	All	HES eligible	All	HES eligible	All	HES eligible
Practices, N	136	135	576	557	712	692
Patients, N	458	191	983	942	1441	1133
Entered study 1990–2000	148	62	484	466	632	528
Entered study 2000–2010	191	87	321	306	512	393
Entered study 2010–2021	119	42	178	170	297	212
Mean years of follow‐up	10.9	11.5	10.3	10.4	10.5	10.6
Steroid users, N	236	107	488	469	724	576
Spinal surgery, N	33	33	209	209	242	242
Ventilation, N	46	46	311	311	357	357
Cardiomyopathy, N	52	24	168	164	220	188

Abbreviation: HES, Hospital Episode Statistics.

While 135 of the 136 recorded practices of the 458 CPRD GOLD patients were eligible for linkage, 265 of these patients (58%) did not have a recorded practice ID and thus could not be determined if they were eligible for linkage. These patients were similar to the GOLD patients who were included in terms of proportions experiencing each event, and median ages at the onset of these events, so excluding patients who were not eligible for linkage still gives representative results for the whole UK population. In contrast, 100% of the Aurum patients had a practice ID recorded, and 557 of these 576 practices were eligible for HES linkage. Around half of the HES eligible patients in GOLD and Aurum had at least one recorded use of corticosteroids, and similar proportions in the two groups had experienced cardiomyopathy and spinal surgery. The majority of corticosteroid use was observed to be initiated before the age of 18 (537/576, 93%). In patients with follow‐up beyond the age of 18 and after 2010, spinal surgery was recorded for 33.5% of patients, ventilation for 49.8%, and cardiomyopathy for 25.7%.

**FIGURE 2:**
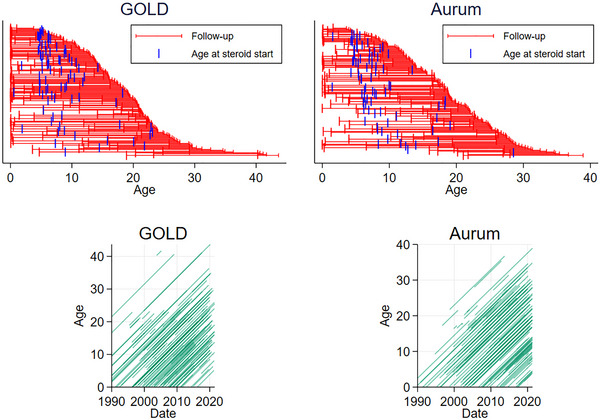
Random sample of ages, ages at first corticosteroid use, and ages against calendar time of 300 patients from the Clinical Practice Research Datalink (CPRD) (150 each from GOLD/Aurum) from 1990 onward.

Follow‐up for a (random) subset of the patients in GOLD and Aurum is plotted in Figure 2.

In general, older patients have later registration dates: when these patients were younger, fewer practices were registered with the CPRD and even fewer with HES linkage. This motivates the left‐truncation at the 1990/practice registration date, since otherwise these older patients are backdated and contribute years to follow‐up that patients who die before they would have joined a practice are not able to contribute, thus inflating estimates of survival (known as immortal time bias) (Tyrer et al., 2022).

Corticosteroid use in general starts before the age of 10, with some instances of corticosteroid use being initiated in patients’ teens and, very rarely, 20s. There is also some evidence of corticosteroid use being backdated; 94 of the 1121 patients’ corticosteroid records begin before their first registration date.

### 3.2 Survival and mortality

**FIGURE 3:**
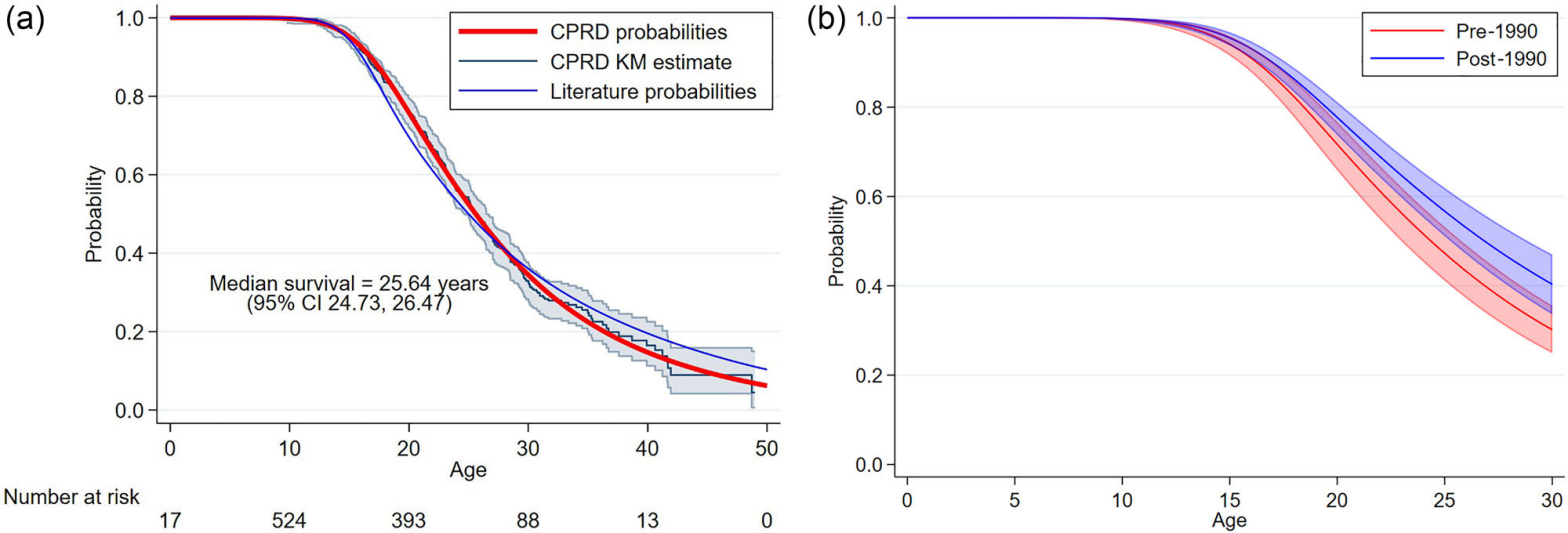
Flexible parametric predictions of (a) survival in the Clinical Practice Research Datalink (CPRD) cohort, and (b) changes in survival over time, left‐truncated at practice registration dates. Also overlain for the whole cohort are Kaplan–Meier (KM) estimates and estimates from a review of international studies (Broomfield et al., 2021). CI, confidence interval.

Survival probabilities across the age range of the patients are shown in Figure 3. The survival probabilities are presented for the whole cohort and also compared between patients born before 1990 to those born after 1990. These are shown up to 30 years to prevent the need for extrapolation beyond observed follow‐up for patients born after 1990.

The Kaplan–Meier curve in Figure 3a ends at 50 years due to the misdiagnosis exclusion criteria. A median survival of 25.6 years (24.7, 26.5) in the whole population was estimated, which is broadly in line with the published literature over the same time period (Broomfield et al., 2021; Landfeldt et al., 2020), although at the lower end of these published estimates. Restricted mean survival time from the flexible parametric model with a lifetime horizon of 50 years was estimated to be 27.9 years (26.8, 29.0). The number at risk does not always decrease over time due to the delayed entry of many patients. Predictions beyond 40 years should be interpreted with caution due to the extremely low patient numbers.

There is a distinct improvement in survival observed over time; patients born before 1990 have a median survival of 23.5 years (22.5, 25.4), while patients born after 1990 have a median survival of 26.5 years (25.2, 27.9). The estimates from this more recent cohort are in line with more recently published estimates of median survival (Broomfield et al., 2021; Landfeldt et al., 2020). This improvement in time is likely to be due to the improvement of integrated, multi‐disciplinary standards of care through measures such as steroid use, ventilatory support, early initiation of cardiac agents, and nutritional assistance. Restricted mean survival time has also increased from 26.9 years from birth (25.5, 28.2) in patients born before 1990 to 29.6 years from birth (27.6, 31.6) in patients born after 1990. There does not appear to be any significant variation in this improvement over time across the 13 different regions of the United Kingdom recorded by CPRD, though patient numbers split across these regions and the two cohorts are quite low.

**FIGURE A1:**
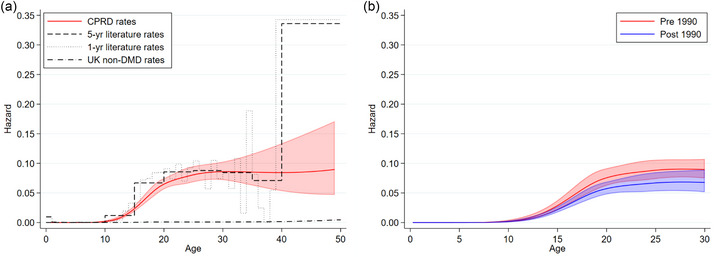
Mortality rates in the Clinical Practice Research Datalink (CPRD) cohort, with overlain estimates from a review of international studies (Broomfield et al., 2021) and from the UK general population, and changes in these rates over time.

Mortality rates across the age range of the patients are shown in Figure A1. These are presented across the entire cohort and over calendar time. Mortality rates in this population are comparable to those in the published literature in a global population (Broomfield et al., 2021) for patients aged 0−40.

### 3.3 Secondary events

**FIGURE 4:**
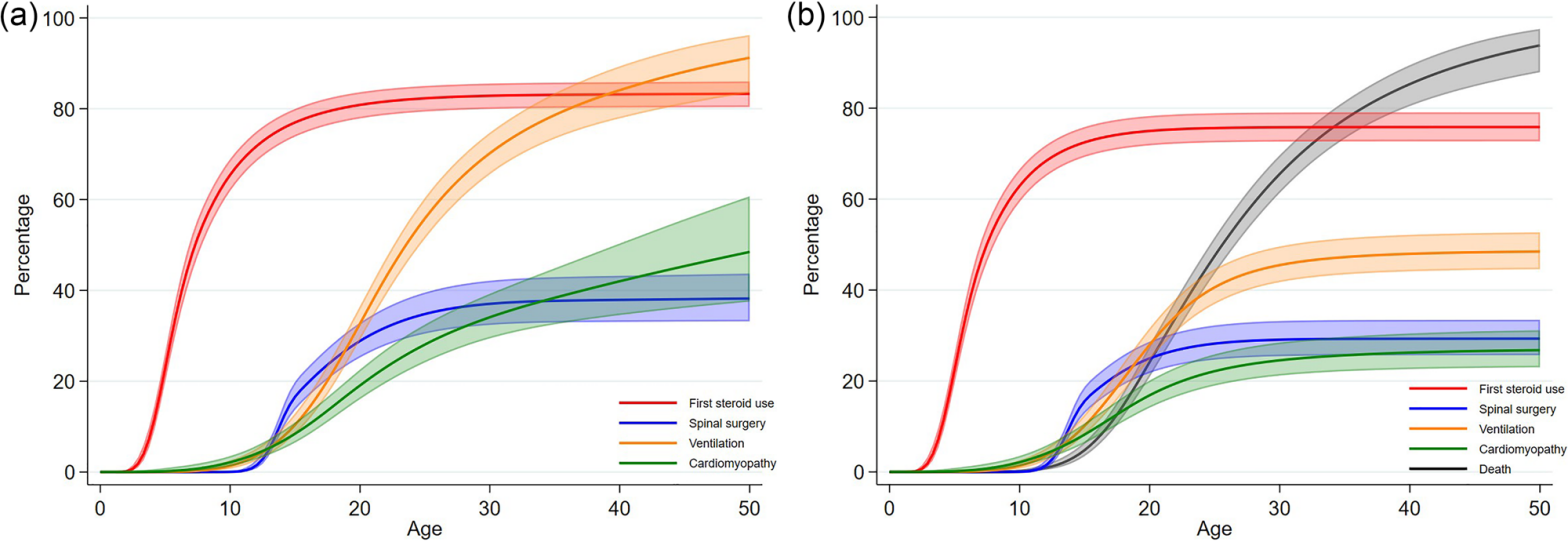
(a) Net probabilities and (b) cumulative incidence functions of experiencing clinical milestones if patients have not yet died and before they die, respectively.

Table A1 contains median and restricted mean estimates of ages at which patients first used corticosteroids, underwent spinal surgery, received ventilation support, and developed a cardiomyopathy (among patients who experienced each event). Probabilities of experiencing each of these events are shown in Figure 4. Net probabilities (probabilities that patients experience each event conditional on reaching a certain age) are presented in Figure 4a. Cumulative incidence functions (CIFs) (probabilities assuming patients may die before experiencing events) are presented in Figure 4b.

In general, patients were first prescribed corticosteroids, then experienced spinal surgery and initiated ventilatory support, and then cardiomyopathy. This is overall consistent with standards of care, which recommend first steroid prescription before the age of 7 years (Birnkrant et al., 2018). However, these events are not clinically related and can happen in any order. Spinal surgery, if it occurred in patients, was likely to occur in their late teens. Around 27% of patients have a recorded diagnosis of cardiomyopathy before dying, around 29% of patients underwent spinal surgery before dying and around 76% of patients were prescribed corticosteroids at least once in their lifetime before dying (Figure 4b). The net probabilities are higher than the CIFs since they are conditioned on the fact that patients survive to a given age, whereas the CIFs allow for the competing event of death. Steroid use was much more common and was initiated at a younger median age in the post‐1990 cohort. On average, age at last corticosteroid prescription was 13.7 years. There was a mean range of 5.2 years between a patient's first and last steroid record in GOLD, with a maximum of 19.6 years; the mean range in Aurum was 5.7 years, with a maximum of 22.8 years.

Table 2 details the prevalence of secondary events across pediatric and adult populations (under/over 18 years).

**TABLE 2:** Prevalence of intermediate events in children and adults with Duchenne muscular dystrophy (DMD).

Milestone	No. of events (%) patients under 18	No. of events (%) patients over 18
First steroid use	323	252
	(63.2)	(41.3)
Spinal surgery	36	206
	(7.1)	(33.8)
Ventilation	61	292
	(11.9)	(47.9)
Cardiomyopathy	31	155
	(6.1)	(25.4)

Steroid use was more common in the pediatric cohort, while the other secondary events all increased in prevalence in the adult population. In adult patients with follow‐up recorded after 2010, the prevalence of spinal surgery, ventilation, and cardiomyopathy also increased. Further breakdown on the prevalence of these events within adult patients was not feasible due to the low patient numbers in this cohort, with only 101 patients living beyond the age of 30.

## 4 DISCUSSION

This study has presented survival probabilities and mortality rates of patients with DMD in the UK population across their lifetime, alongside equivalent estimates of secondary clinical milestones. It is consistent with analysis of a global population (Broomfield et al., 2021) and provides a source of external mortality data for natural history modeling of DMD patients in the United Kingdom. It presents a clinical overview of patients with DMD in primary and secondary care in the United Kingdom. The CPRD is also being used in ongoing analysis to estimate the healthcare resource utilization of DMD (linked with HES statistics) (Morgan et al., 2021) and to investigate the health of mothers of patients with DMD (Fraser et al., 2020). Previous work identified the CPRD as a useful tool for identifying rare diseases and quantifying their burden (Maguire et al., 2017). Rare disease‐specific registry databases are an area of future interest, facilitating confirmed diagnoses, regular and disease‐specific longitudinal covariate information, and more efficient natural history modeling, mirroring registries in cancer and Covid‐19 (Wood et al., 2021).

A large population of 1121 male patients was identified, 337 of whom had died, with 10.6 years of follow‐up per patient on average. Median life expectancy was estimated to be 25.64 years (24.73, 26.47), which is consistent with literature reviews from populations containing follow‐up at the same time (Broomfield et al., 2021; Landfeldt et al., 2020). Improvements in survival in the post‐1990 cohort were observed, which is similarly consistent with literature findings as standards of care have improved, most notably due to increased corticosteroid use, early prescription of cardiac agents, and the introduction of assisted ventilation (Bushby et al., 2010a, 2010b).

Results on secondary events should be interpreted cautiously since patients in the CPRD are not under observation throughout their lifetimes. Patients initiated corticosteroid use at a median age of 6.06 years (5.77, 6.29), although there are a proportion of patients who never begin corticosteroid use. While corticosteroids have been used in DMD for over 30 years, their routine prescription is more recent, so older patients may not have been prescribed them (Bushby et al., 2010a, 2010b). Alternatively, a plateau in disease progression (Bushby et al., 2010a, 2010b), or an adverse reaction that may have occurred before the patient moved to a practice that recorded CPRD data, may lead to a lack of corticosteroid prescription in patient records. Some observations of steroid initiation may be misclassified as later in a patient's life, if earlier steroid initiation was not recorded either through error or due to the patient not being at a CPRD‐recording practice. The use of corticosteroids after loss of ambulation/in adults has only increased in recent years too, due to the suggestion of a beneficial effect on upper limb and respiratory and cardiac functions (Bushby et al., 2010a, 2010b). Future work could focus on age at last corticosteroid prescription to gain inference on steroid stopping in older DMD populations, since the limitations in recording of corticosteroid prescription in the CPRD mean the results may not fully reflect clinical practice.

Patients underwent spinal surgery at a median age of 14.79 (14.29, 15.09) and the median age of cardiomyopathy diagnosis was 15.26 (14.22, 16.70). Spinal surgery was observed to occur at a mean age of 14 years in a previous UK population of 100 boys (Eagle et al., 2007). In this study, the prevalence of spinal surgery was 47% (Eagle et al., 2007), which is notably higher than our study. However, it is possible that patients in our study received spinal surgery while not under observation at a CPRD‐consenting practice. Moreover, there is a significant proportion of young patients in our dataset (15% below age 10). Spinal surgery prevalence may also have decreased since then, due to more frequent and earlier initiation of corticosteroids. The same is true of ventilatory support, which was initiated at a median age of 17.0 years (16.5, 18.30).

Cardiomyopathy was also less prevalent in our study than it has previously been reported (Spurney, 2011). This could be explained by several factors. First, the same issues affecting spinal surgery records may also affect cardiomyopathy records. There is also no harmonized agreement on the definition of cardiomyopathy in DMD; the most standard definition is impaired left ventricular function; however, the cut‐off values to defined left ventricular impairment vary from clinic/clinician to clinic/clinician. As previously mentioned regarding spinal surgery, a significant proportion (15%) of patients in the cohort were younger than 10 years of age, and cardiomyopathy is not expected to become manifest before this age. Through the inspection of patient records for cardiac magnetic resonance imaging, diagnosis rates may also increase in the future in the young cohort. Cause of death information could also increase the observed proportion of cardiomyopathy diagnoses, although in patients with DMD, the primary cause of death is almost invariably listed as “Muscular Dystrophy” (ICD‐9 code 359.1/ICD‐10 code G71.0).

Among patients living to 30 years, there was a 37% (32%, 42%) probability of requiring spinal surgery. The probability of a patient with DMD who has survived to age 30 experiencing cardiomyopathy is 34% (29%, 39%), increasing to 42% (34%, 50%) if they survive to 40.

Assumptions were made relating to the coding of dates of birth, cut‐offs for implausible dates and clinically implausible ages, and accuracy of diagnosis. These were varied in sensitivity analyses (Table A2) and had minimal impact on results. In particular, alternative assumptions about diagnosis criteria gave similar results to analysis on the full cohort. The possibility of confirming diagnoses of DMD in the CPRD through free‐text searches could be explored (Maguire et al., 2017).

The CPRD, as a retrospective database, is also subject to different factors that may limit data accuracy as well as comparability with future study populations. Non‐compliance of treatments such as steroid usage, cardiac medications, and even assisted ventilation are pertinent examples. The availability of different corticosteroid doses (see Tables A3 and A4) may augment this. Additionally, diagnostic variability exists beyond the discussed example of cardiomyopathy, such as in pulmonary function, which feeds into ventilation initiation. This lack of standardization limits comparability between the entire UK population covered by the CPRD and future studies in specific populations where diagnoses of these intermediate events may be standardized. Comparability is further hindered by the phenotypic variability of DMD (Birnkrant et al., 2022), where survival may be dependent on genetic modifiers that are different in smaller study populations. The consistency of survival estimates in the CPRD with published literature suggests that this possibility is not specific to retrospective electronic health records. However, it remains important to note that the CPRD should not be used as a control for a future clinical study without thorough verification of population comparability between the two, including appropriate use of the CPRD as a historical control.

These findings confirm the use of the CPRD as a genuine population measure for mortality in patients with DMD in the United Kingdom across the timeframe of the study, and as a future source for other rare diseases. Mortality data collection should always be considered while designing natural history studies and models to gain accurate, unbiased, and uninflated estimates of intermediate disease progression. This analysis provides a source in the UK general population that is consistent with global populations (Broomfield et al., 2021; Landfeldt et al., 2020). However, comparability between the CPRD population and a population from a novel treatment (where standards of care and treatment adherence may differ) should be ensured to avoid falsely beneficial effects—though this is not a limitation specific to the CPRD. Comparability of intermediate events with published estimates was also limited, and this study instead provides a descriptive overview of these events. Finally, this study provides a framework for the analysis of mortality in other rare diseases to facilitate natural history modeling.

## AUTHOR CONTRIBUTIONS

**Jonathan Broomfield**: Conceptualization; methodology; software; data curation; investigation; validation; formal analysis; project administration; visualization; funding acquisition; writing—original draft; writing—review and editing; resources. **Keith Abrams**: Conceptualization; methodology; supervision; writing—review and editing; funding acquisition; visualization. **Nick Latimer**: Methodology; writing—review and editing; visualization; supervision. **Michela Guglieri**: Supervision; validation; writing—review and editing. **Mark Rutherford**: Methodology; formal analysis; validation; visualization; writing—review and editing; supervision. **Michael Crowther**: Conceptualization; methodology; writing—review and editing; visualization; funding acquisition; supervision.

## CONFLICT OF INTEREST STATEMENT

Jonathan Broomfield, Keith Abrams and Michael Crowther have received consultancy fees from Duchenne UK. Michela Guglieri has received research funding from Sarepta, PTC, Muscular Dystrophy UK, NIH and H2020; is (or has been) chief investigator/principal investigator for clinical trials of Pfizer, Italfarmaco, Santhera, Roche, ReveraGen, Dynacure, Dyne; is a member of Advisory boards for Pfizer, NS Pharma, Dyne (honoraria through Newcastle University); and has received speaker honoraria from Sarepta, Italfarmaco, Novartis and Roche.

### PEER REVIEW

The peer review history for this article is available at https://publons.com/publon/10.1002/brb3.3331.

## Data Availability

This study is based in part on data from the Clinical Practice Research Datalink obtained under license from the UK Medicines and Healthcare products Regulatory Agency. The data are provided by patients and collected by the NHS as part of their care and support. The interpretation and conclusions contained in this study are those of the authors alone. Copyright © [2022], re‐used with the permission of The Health & Social Care Information Centre. All rights reserved. Researchers who wish to access CPRD data must submit a protocol to the CPRD research committee at www.erap.cprd.com.

## CPRD data extraction

A protocol was approved by the CPRD in July 2021 and is available at https://cprd.com/protocol/age‐specific‐mortality‐rates‐males‐diagnosed‐duchenne‐muscular‐dystrophy. Both data from CPRD GOLD and Aurum were extracted; the only difference in these databases is the software used by practices to store data. This leads to some minor differences between the two sources in terms of structure and coding; for instance, the CPRD GOLD dataset contains a variable denoting the date at which data from each practice are of “research quality” (denoted the “up to standard” date) regarding practice death recording and data gaps, whereas the Aurum dataset does not. The patients visiting GOLD and Aurum are in theory no different.

See FigureA1

A feasibility count in CPRD GOLD identified 458 male patients with the medical code 5393 (read code F391000) “Duchenne muscular dystrophy.” A count in CPRD Aurum identified 985 male patients using the medical codes 127307018, 3746961000006114, and 905831000006110. Patients did not need to be currently registered at their practice, and all follow‐ups were considered (not just follow‐up classed by a CPRD‐derived algorithm as “up‐to‐standard”).

Date of birth was calculated as follows. For patients over 16, only year of birth was provided, so date of birth was calculated as “01/07/year of birth,” or as the date of the first recorded visit if this was before “01/07/year of birth” but within a year of this date. For patients under 16, year and month of birth were provided; thus, “15/month of birth/year of birth” was used instead. In the sensitivity analysis, patients were first assumed to be born at the beginning of the respective period (i.e., “01/01/year of birth” or “01/month of birth/year of birth”) and then at the end of the respective period (“31/12/year of birth” or “28, 29, 30, 31/month of birth/year of birth”).

Spinal surgery in patients was identified from HES records as the first recorded OPCS code beginning V22 up to V70, with the exception of any code beginning V55 (since these identify the spine level of the surgery, not a distinct spinal operation). The age of patients at spinal surgery was taken as the midpoint of their age at the start date and end date of the episode; this is a mild assumption, since these two dates are ordinarily just a few days apart. Ventilation support was identified in the same way, except using the OPCS codes beginning E85. Cardiomyopathy in GOLD patients was identified using the first recorded medcode of codes 3204, 22993, and 44272, and similarly in Aurum patients using the medcodes 142397010, 300054017, 300062013, and 460126017.

There is a possibility that some patients included in the final analysis do not have DMD. Multiple patients were observed to live into their sixth decade; while this has been observed in isolated instances, it is not yet as commonplace as the raw CPRD data would suggest. Our initial inclusion criteria identified 12 patients out of 1133 (1.1%) above the age of 50, which was implausibly high. Additionally, these medcodes identified 324 females in a CPRD feasibility count, when the global prevalence of DMD in females is in fact estimated to be much lower. One possibility of this high count could be if the patients are female relatives of male patients with DMD, who are asymptomatic carriers of the mutation. We therefore implied a further exclusion of removing patients above the age of 50. While this is not ideal, the consistency of the results with other published literature (in which DMD diagnosis was confirmed genetically and misdiagnoses far less plausible)—and the lack of variation in results from the sensitivity analyses—supports the diagnosis definition used, and at least suggests that the inclusion of possible misdiagnoses appears to have minimal impact on the results.

## A2 Mortality rates

The flexible parametric approach used in this study provides a more plausible smooth increase in mortality over this period. Patient numbers above age 40 are low from the literature and the CPRD (*n* = 13, Figure 3a) explaining the disagreement after this age. The widening confidence interval is a reflection of the small patient numbers contributing to the hazard as age increases, and the disagreement between the estimates at these ages is not too concerning given the low patient numbers.

## A3 Secondary events

See Table A1

**TABLE A1:** Summary statistics of ages at clinical milestones of Duchenne muscular dystrophy (DMD).

Milestone	No. of events (%)	Median (95% CI)	Mean (95% CI)
First steroid use	479	6.06	6.34
	(46.7)	(5.77, 6.29)	(6.11, 6.58)
Spinal surgery	155	14.79	14.68
	(15.0)	(14.29, 15.09)	(14.34, 15.01)
Ventilation	286	16.97	18.12
	(27.1)	(16.50, 18.31)	(17.38, 18.87)
Cardiomyopathy	133	15.26	15.17
	(12.5)	(14.22, 16.70)	(14.08, 16.27)

Abbreviation: CI, confidence interval.

## A4 Sensitivity analyses

Table A2 displays median survival times, restricted mean survival times and time to first corticosteroid use (in years), and mortality rates at 10 and 20 years (in person‐years) across the different scenarios considered in the sensitivity analysis. These scenarios were assuming a lower bound and upper bound for dates of birth, including patients who were observed to be older than 50, restricting analysis to patients with a Muscular Dystrophy ICD‐10 code in their HES records, additional left truncation at up‐to‐standard date (for GOLD patients), left truncation at 2000 or 2010 (instead of 1990) for all patients, inflated mortality rates after 50 years, and restricting analysis to patients with more than one diagnosis of DMD in their CPRD records. They are compared to the base case analysis.

**TABLE A2:** Results of sensitivity analyses by scenario.

Scenario	Median survival	RMST	10‐Year mortality rate	20‐Year mortality rate	Median first steroid
Base case	25.6 (24.6, 26.5)	27.9 (26.8, 29.0)	0.0016 (0.0007, 0.0036)	0.065 (0.056, 0.075)	6.1 (5.8, 6.3)
Lower bound DOB	25.1 (24.2, 26.0)	27.5 (26.3, 28.6)	0.0021 (0.0010, 0.0043)	0.067 (0.059, 0.077)	5.8 (5.6, 6.0)
Upper bound DOB	26.0 (25.1, 27.0)	28.3 (27.2, 29.4)	0.0013 (0.0005, 0.0030)	0.062 (0.053, 0.072)	6.3 (5.9, 6.5)
Including age >50	25.6 (24.6, 26.5)	28.4 (27.3, 29.5)	0.0017 (0.0008, 0.0037)	0.066 (0.057, 0.076)	6.1 (5.8, 6.3)
HES ICD record	25.4 (24.4, 26.5)	27.9 (26.8, 29.0)	0.0015 (0.0006, 0.0036)	0.067 (0.058, 0.078)	6.2 (5.8, 6.4)
Left truncation at UTS	25.5 (24.6, 26.5)	27.8 (26.7, 28.9)	0.0017 (0.0008, 0.0037)	0.065 (0.057, 0.075)	5.9 (5.7, 6.3)
Left truncation at 2000	25.6 (24.6, 26.5)	27.8 (26.7, 28.9)	0.0018 (0.0008, 0.0041)	0.064 (0.055, 0.074)	5.8 (5.6, 6.1)
Left truncation at 2010	27.0 (25.7, 28.5)	29.1 (27.7, 30.5)	0.0027 (0.0011, 0.0064)	0.049 (0.040, 0.060)	5.3 (5.1, 5.6)
Inflated mortality >50	25.6 (24.6, 26.5)		0.0074 (0.0052, 0.0106)	0.053 (0.046, 0.061)	6.1 (5.8, 6.3)
Multiple DMD diagnoses	25.7 (21.5, 29.6)	28.1 (23.6, 32.6)	0.0021 (0.0002, 0.0217)	0.071 (0.045, 0.114)	5.9 (5.1, 7.3)

Abbreviation: DOB, Date of birth; DMD, Duchenne muscular dystrophy; RMST, Restricted mean survival time; UTS, Up‐to‐standard date.

The different scenarios had very minimal impact on the results. In general, median survival was still estimated to occur between the age of 25 and 26, and mean survival (RMST) between 27 and 29 years, with results from a more recent population (left truncation at 2010) suggesting that survival has improved over time. Mortality rates were comparable across the scenarios, with between 1 and 3 deaths per 1000 person years estimated at 10 years and between 50 and 70 deaths per 1000 person years at 20 years. Median age at first corticosteroid use among those who take corticosteroids was also consistent across different scenarios; it appears that in more recent cohorts, patients are initiating corticosteroid use at a slightly younger age. RMST could not be calculated for the inflated mortality scenario due to software limitations.

## A5 DMD corticosteroid codes

See Table A3 and Table A4.

**TABLE A3:** Duchenne muscular dystrophy (DMD) corticosteroid codes for Clinical Practice Research Datalink (CPRD) GOLD.

Product code	GOLD product name
44	Prednisolone 5 mg gastro‐resistant tablets
95	Prednisolone 5 mg tablets
557	Prednisolone 2.5 mg gastro‐resistant tablets
578	Prednisolone 1 mg tablets
820	Prednisolone 20 mg/100 mL retention enema
955	Prednisolone 5 mg soluble tablets
1063	Prednesol 5 mg tablet (Sovereign Medical Ltd.)
2368	Prednisolone 2.5 mg tablet
2704	Prednisolone 25 mg tablets
2949	Prednisone 5 mg tablets
3557	Prednisone 1 mg tablets
3913	Prednisolone sodium phosphate 5 mg suppositories
3992	Deflazacort 6 mg tablets
5490	Deltacortril 5 mg gastro‐resistant tablets (Alliance Pharmaceuticals Ltd.)
5913	Deltacortril 2.5 mg gastro‐resistant tablets (Alliance Pharmaceuticals Ltd.)
6805	Prednisolone 20 mg/application foam enema
8306	Prednisolone 25 mg/1 mL suspension for injection ampoules
9375	Deflazacort 1 mg tablets
14695	Prednisolone 1.3 mg with cinchocaine 1 mg suppositories
15164	Cinchocaine 1 mg /Prednisolone hexanoate 1.3 mg suppositories
18660	Deltastab 25 mg/1 mL suspension for injection ampoules (AMCo)
19141	Prednisolone 5 mg soluble tablets (AMCo)
20577	Calcort 6 mg tablet (Shire Pharmaceuticals Ltd.)
21417	Prednisolone 5 mg tablets (A A H Pharmaceuticals Ltd.)
22555	Calcort 1 mg tablets (Shire Pharmaceuticals Ltd.)
27962	Deltastab 1 mg tablet (Waymade Healthcare Plc)
28375	Prednisolone 2.5 mg gastro‐resistant tablets (A A H Pharmaceuticals Ltd.)
28376	Prednisolone 2.5 mg Gastro‐resistant tablet (Biorex Laboratories Ltd.)
28859	Deltastab 5 mg tablet (Waymade Healthcare Plc)
29333	Prednisolone 5 mg tablets (Actavis UK Ltd.)
31327	Prednisolone steaglate 6.65 mg tablet
31532	Prednisolone 5 mg gastro‐resistant tablets (A A H Pharmaceuticals Ltd.)
32803	Prednisolone 5 mg gastro‐resistant tablets (Actavis UK Ltd.)
32835	Prednisolone 5 mg tablets (Wockhardt UK Ltd.)
33691	Prednisolone 5 mg gastro‐resistant tablet (Biorex Laboratories Ltd.)
33988	Prednisolone 5 mg tablet (Co‐Pharma Ltd.)
33990	Prednisolone 5 mg tablet (IVAX Pharmaceuticals UK Ltd.)
34109	Prednisolone 5 mg gastro‐resistant tablet
34221	Prednisolone suppositories
34393	Prednisolone 5 mg gastro‐resistant tablets (Teva UK Ltd.)
34404	Prednisolone 1 mg tablets (Actavis UK Ltd.)
34452	Prednisolone 1 mg tablets (A A H Pharmaceuticals Ltd.)
34461	Prednisolone 2.5 mg gastro‐resistant tablets (Actavis UK Ltd.)
34631	Prednisolone 1 mg tablet (Co‐Pharma Ltd.)
34660	Prednisolone 1 mg tablets (Kent Pharmaceuticals Ltd.)
34748	Prednisolone 1 mg tablets (Teva UK Ltd.)
34781	Prednisolone 5 mg tablets (Kent Pharmaceuticals Ltd.)
34914	Prednisolone 1 mg tablet (Celltech Pharma Europe Ltd.)
34978	Prednisolone 1 mg tablets (Wockhardt UK Ltd.)
38407	Prednisolone 20 mg tablet
41335	Calcort 6 mg tablets (Sanofi)
41515	Prednisolone 5 mg tablets (Teva UK Ltd.)
41745	Prednisolone 25 mg tablets (Zentiva)
42408	Prednisolone 40 mg/100 mL enema
43544	Prednisone 5 mg Tablet (Knoll Ltd.)
44380	Prednisone 1 mg modified‐release tablets
44723	Prednisone 5 mg modified‐release tablets
45302	Prednisolone 5 mg tablet (Biorex Laboratories Ltd.)
46711	Prednisone 2 mg modified‐release tablets
47142	Prednisolone 5 mg soluble tablet (Amdipharm Plc)
48320	Prednisolone 20 mg/100 mL enema standard tube
51753	Prednisolone 1 mg tablets (Strides Shasun (UK) Ltd.)
53313	Prednisolone 20 mg/5 mL oral suspension
53336	Prednisolone 25 mg tablets (A A H Pharmaceuticals Ltd.)
54118	Prednisolone 25 mg/5 mL oral suspension
54434	Prednisolone 2.5 mg/5 mL oral suspension
55024	Prednisolone 5 mg/5 mL oral solution
55480	Prednisolone 2.5 mg gastro‐resistant tablets (Alliance Pharmaceuticals Ltd.)
56891	Prednisolone 1 mg tablets (Waymade Healthcare Plc)
58000	Prednisolone 5 mg tablets (Almus Pharmaceuticals Ltd.)
58061	Prednisone 50 mg tablets
58234	Prednisolone 10 mg/5 mL oral solution
58369	Prednisolone 5 mg tablets (Boston Healthcare Ltd.)
58384	Prednisolone 1 mg tablets (Almus Pharmaceuticals Ltd.)
58987	Prednisolone 5 mg gastro‐resistant tablets (Phoenix Healthcare Distribution Ltd.)
59229	Dilacort 5 mg gastro‐resistant tablets (Auden McKenzie [Pharma Division] Ltd.)
59283	Dilacort 2.5 mg gastro‐resistant tablets (Auden McKenzie [Pharma Division] Ltd.)
59338	Prednisolone 1 mg/5 mL oral solution
59912	Prednisolone 5 mg gastro‐resistant tablets (Waymade Healthcare Plc)
60421	Prednisolone 5 mg tablets (Strides Shasun (UK) Ltd.)
61132	Prednisolone 1 mg tablets (Boston Healthcare Ltd.)
61162	Prednisolone 5 mg tablets (Waymade Healthcare Plc)
61689	Prednisolone 5 mg soluble tablets (A A H Pharmaceuticals Ltd.)
62656	Prednisone 5 mg tablet (Hillcross Pharmaceuticals Ltd.)
63066	Prednisolone 2.5 mg tablets
63082	Prednisolone 20 mg tablets
63172	Prednisolone 10 mg tablets
63214	Prednisolone 5 mg soluble tablets (Alliance Healthcare (Distribution) Ltd.)
63549	Prednisolone 1 mg/mL oral solution (Logixx Pharma Solutions Ltd.)
63791	Prednisolone 5 mg/5 mL oral solution unit dose
64007	Pevanti 10 mg tablets (AMCo)
64008	Pevanti 2.5 mg tablets (AMCo)
64009	Pevanti 20 mg tablets (AMCo)
64128	Pevanti 5 mg tablets (AMCo)
64221	Prednisolone 5 mg/5 mL oral suspension
64416	Prednisolone 10 mg/mL oral solution sugar free
65020	Prednisolone 25 mg/5 mL oral solution
65626	Prednisolone 10 mg/5 mL oral suspension
66015	Prednisolone Dompe 5 mg/5 mL oral solution unit dose (Logixx Pharma Solutions Ltd.)
66550	Prednisolone 5 mg gastro‐resistant tablets (Alliance Healthcare [Distribution] Ltd.)
66633	Prednisolone 20 mg/application foam enema (Alliance Healthcare [Distribution] Ltd.)
66645	Prednisolone 5 mg/5 mL oral solution unit dose (Logixx Pharma Solutions Ltd.)
66914	Prednisolone 1 mg gastro‐resistant tablets
67076	Prednisolone 20 mg/5 mL oral solution
67107	Prednisolone 5 mg gastro‐resistant tablets (Alliance Pharmaceuticals Ltd.)
67507	Prednisolone 30 mg tablets
67559	Prednisolone 5 mg/5 mL oral solution unit dose (A A H Pharmaceuticals Ltd.)
68130	Prednisolone sodium phosphate 5 mg suppositories (A A H Pharmaceuticals Ltd.)
68497	Prednisolone 2.5 mg gastro‐resistant tablets (Waymade Healthcare Plc)
69568	Dilacort 5 mg gastro‐resistant tablets (Crescent Pharma Ltd.)
69678	Prednisolone 20 mg/application foam enema (Essential Generics Ltd.)
69686	Pevanti 25 mg tablets (AMCo)
69811	Prednisolone 30 mg tablets (Actavis UK Ltd.)
70603	Prednisolone 5 mg soluble tablets (Focus Pharmaceuticals Ltd.)

**TABLE A4:** Duchenne muscular dystrophy (DMD) corticosteroid codes for Clinical Practice Research Datalink (CPRD) Aurum.

Product code	Aurum product name
210841000033110	Calcort 6 mg tablets (Sanofi)
428941000033110	Deflazacort 6 mg tablets
431541000033114	Deltacortril 2.5 mg gastro‐resistant tablets (Phoenix Labs Ltd.)
431641000033110	Deltacortril 5 mg gastro‐resistant tablets (Phoenix Labs Ltd.)
1114241000033110	Prednisolone 2.5 mg gastro‐resistant tablets
1114341000033110	Prednisolone 5 mg gastro‐resistant tablets
1127341000033110	Prednisolone 5 mg soluble tablets
1130741000033110	Prednisolone 25 mg tablets
1131741000033110	Prednisolone 1 mg tablets
1131841000033110	Prednisolone 2.5 mg tablets
1131941000033110	Prednisolone 5 mg tablets
1132141000033110	Prednisone tablets 5 mg
1137341000033110	Prednesol soluble tablets 5 mg
1581041000033110	Calcort 30 mg tablets (Shire Pharmaceuticals Ltd.)
1601341000033110	Deflazacort 30 mg tablets
1601441000033110	Deflazacort 1 mg tablets
10212141000033100	Prednisolone 10 mg tablets
10212341000033100	Prednisolone 20 mg tablets
10266841000033100	Prednisolone 5 mg/5 mL oral solution unit dose
10494341000033100	Prednisolone 10 mg/mL oral solution sugar free
